# Stochastic modeling and simulation of reaction-diffusion system with Hill function dynamics

**DOI:** 10.1186/s12918-017-0401-9

**Published:** 2017-03-14

**Authors:** Minghan Chen, Fei Li, Shuo Wang, Young Cao

**Affiliations:** Department of Computer Science, Virginia Tech, Blacksburg, 24061 VA USA

**Keywords:** Reaction diffusion master equation (RDME), Hill function, Stochastic simulation, Hybrid method

## Abstract

**Background:**

Stochastic simulation of reaction-diffusion systems presents great challenges for spatiotemporal biological modeling and simulation. One widely used framework for stochastic simulation of reaction-diffusion systems is reaction diffusion master equation (RDME). Previous studies have discovered that for the RDME, when discretization size approaches zero, reaction time for bimolecular reactions in high dimensional domains tends to infinity.

**Results:**

In this paper, we demonstrate that in the 1D domain, highly nonlinear reaction dynamics given by Hill function may also have dramatic change when discretization size is smaller than a critical value. Moreover, we discuss methods to avoid this problem: smoothing over space, fixed length smoothing over space and a hybrid method.

**Conclusion:**

Our analysis reveals that the switch-like Hill dynamics reduces to a linear function of discretization size when the discretization size is small enough. The three proposed methods could correctly (under certain precision) simulate Hill function dynamics in the microscopic RDME system.

## Background

Cell reproduction requires elaborate spatial and temporal coordination of crucial events, such as DNA replication, chromosome segregation, and cytokinesis. In cells, protein species are well organized and regulated throughout their life cycles. Theoretical biologists have been using classic chemical reaction rate laws with deterministic ordinary differential equations (ODEs) and partial differential equations (PDEs) to model molecular concentration dynamics in spatiotemporal biological processes. However, wet-lab experiments in single cell resolution demonstrate that biological data present considerable variations from cell to cell. The variations arise from the fact that cells are so small that there exist only one or two copies of genes, tens of mRNA molecules and hundreds or thousands of protein molecules [1–3]. At this scale, the traditional way of modeling molecule “concentration” is not applicable. Noise in molecule populations cannot be neglected, as noise may play a significant role in the overall dynamics inside a cell. Therefore, to accurately model the cell cycle mechanism, discrete and stochastic modeling and simulation should be applied.

A convenient strategy to build a stochastic biochemical model is to break a deterministic model into a list of chemical reactions and simulate them with Gillespie’s stochastic simulation algorithm (SSA) [4, 5]. One of the major difficulties in this conversion strategy lies in the propensity calculation of reactions. Gillespie’s SSA is well defined for mass action rate laws. However, in many biochemical models, in addition to mass action rate laws, other phenomenological reaction rate laws are often used. For example, the Michaelis-Menten equation [6] and Hill functions [7] are widely used in biological models to model the fast response to signals in regulatory control systems. Although theoretically these phenomenological rate laws may be generated from elementary reactions with mass action rate laws, in practice the detailed mechanisms behind these phenomenological rate laws are not well known and may not be very important. Stochastic modeling and simulation with these phenomenological rate laws are sometimes inevitable.

In recent years, stochastic modeling and simulation for spatiotemporal biological systems, particularly reaction-diffusion systems, have captured more and more attention. Several algorithms and tools [8–11] to model and simulate reaction-diffusion systems have been proposed. These methods can be categorized into two theoretical frameworks: the spatially and temporally continuous Smoluchowski modeling framework [12] and the compartment-based modeling framework, formulated as the spatially discretized reaction-diffusion master equation (RDME) [13, 14]. The Smoluchowski framework [12, 15, 16] stores the exact position of each molecule and is mathematically fundamental, whereas the RDME is coarse-grained and better suited for large scale simulations [17]. In RDME, the spatial domain is discretized into small compartments. Within each compartment, molecules are considered “well-stirred”. Under the RDME scheme, diffusion is modeled as continuous time random walk on mesh compartments, while reactions fire only among molecules in the same compartment. Stochastic dynamics of the chemical reactions in each compartment is governed by the chemical master equation (CME) [18, 19]. Yet, the CME is computationally impossible to solve for most practical problems. Stochastic simulation methods were then applied to generate realizations of system trajectories. It has been well established that the discretization compartment size for RDME should be smaller than the mean free path of the reactions for the compartment to be considered as well-stirred [20]. In addition, it has been proved that the RDME of bi-molecular reactions in 3D domain becomes incorrect and yields unphysical results when the discretization size approaches microscopic scale [21–23].

In this paper, we focus on the stochastic modeling of reaction-diffusion systems with reaction rate laws given by Hill functions. In the Results section, we present our numerical analysis on a toy model of reaction-diffusion system with Hill function dynamics. We will show that the RDME framework of the Hill function dynamics has serious simulation defects when the discretization size approach microscopic limit: When the discretization size is small enough, the typical switching pattern of Hill dynamics becomes linear to the input signal (and the discretization size). Later, we propose potential solutions for the discretization of the reaction-diffusion systems with Hill function rate laws. Finally, we conclude this paper with a discussion on RDME for general nonlinear functions and the hybrid method.

### Caulobacter modeling

*Caulobacter crescentus* captures great interest in the study of asymmetric cell division. When a *Caulobacter* cell divides, it produces two functionally and morphologically distinct daughter cells. The asymmetric cell division of *Caulobacter crescentus* requires elaborate temporal and spatial regulations [24–27]. In literature [28–30], four essential “master regulators” of the *Caulobacter* cell cycle, DnaA, GcrA, CtrA and CcrM, have been identified. These master transcription regulators determine the dynamics of around 200 genes. They oscillate temporally to drive the dynamics of cell cycle. Among them, the molecular mechanisms governing CtrA functions have been well studied. The simulation we are concerned with in this paper is also related to this CtrA module. So we give a brief introduction to it.

In swarmer cells, a two-component phosphorelay system (with both CckA and ChpT) phosphorylates the CtrA. Then the chromosomal origin of replication (*Cori*) is bound by the phosphorylated CtrA (CtrAp) to inhibit the initiation of chromosome replication [31]. Later during the swarmer-to-stalked transition period, CtrAp gets dephosphorylated and degraded, allowing the initiation of chromosome replication again. Thus the CtrA has important impact on the chromosome replication in our model, and should be well regulated.

The regulation of CtrA is achieved by the histidine kinase CckA through the following pathway. An ATP-dependent protease, ClpXP, degrades CtrA [32, 33] and is localized to the stalk pole by CpdR. As the nascent stalked cell progresses through the cell cycle, CpdR is phosphorylated by CckA/ChpT, losing it polar localization, and consequently losing its ability to recruit ClpXP protease for CtrA degradation. In addition, CtrA is reactivated through CckA/ChpT phosphorylation [34]. Moreover, the regulatory network of the histidine kinases CckA is influenced by a non-canonical histidine kinase, DivL [35]. DivL promotes CckA kinase, which then phosphorylates and activates CtrA in the swarmer cell. During the swarmer-to-stalked transition period, DivL activity is down-regulated, thereby inhibiting CckA kinase activity. As a result, dephosphorylation and degradation of CtrA trigger the initiation of chromosome replication.

**Fig. 1:**
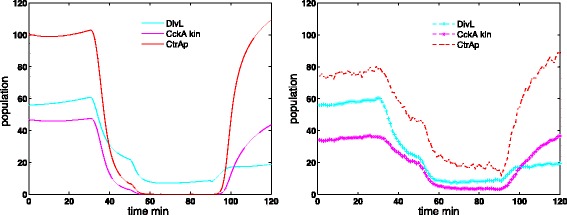
The population oscillation of CtrAp during *Caulobacter crescentus* cell cycle. Left figure shows the simulation result of deterministic model and the right figure shows the stochastic simulation result. In the swamer stage (*t*=0∼30min), the CtrA is phosphorylated and at high population level state, which inhibits the initiation of chromosome replication. During swarmer-to-stalked transition (*t*=30∼50min), the CtrAp population quickly switch to low state, allowing the consequent initiation of chromosome replication in the stalked stage

In order to study the regulatory network in *Caulobacter crescentus*, Subramanian et al. [26, 27] developed a deterministic model with six major regulatory proteins. The deterministic model provides robust switching between swarmer and stalked states. Figure 1 (left) demonstrates the total population change during the *Caulobacter crescentus* cell cycle with this deterministic model. In the swarmer stage (from *t*=0 to 30 min), the CtrA is phosphorylated at a high population level, which inhibits the initiation of chromosome replication. During the swarmer-to-stalked transition period (from *t*=30 to 50 min), the CtrAp population quickly drops to a low level, allowing the consequent initiation of chromosome replication in the stalked stage.

In stochastic simulation of the spatiotemporal model of this regulatory network, the phosphorylated CtrA (CtrAp) population switch from a high level in swarmer stage to a low level in stalked stage is not as sharp as expected, shown in Fig. 1 (right). On the other hand, the DivL population level from the stochastic simulation seems similar to that from the deterministic simulation. A simple analysis suggests that the Hill function dynamics, which models the up regulation of CckA kinase activity by DivL, might be the culprit. Further investigation leads to the discovery of the Hill function limitation at small discretization sizes, as analyzed in the next section.

## Methods

### Reaction diffusion master equation

Before we plunge into Hill functions in reaction-diffusion systems, we will first briefly review mathematical modeling and simulation methods of spatially inhomogeneous stochastic systems.

The dynamics of a spatially inhomogeneous stochastic system has been considered as governed by the reaction-diffusion master equation (RDME), developed in an early work of Gardiner [13]. The RDME framework partitions the spatial domain into small compartments, such that molecules within each compartment can be considered well-stirred. Assume a biochemical system of *N* species {*S*_1_,*S*_2_,…,*S*_*N*_} and *M* reactions within a spatial domain *Ω*, which is partitioned into *K* grids *V*_*k*_, *k*=1,2,…,*K*. For simplicity, we assume that the space *Ω* is one dimensional (1D). Each species population, as well as the reactions in the system will have a local copy for each compartment. The state of the reaction-diffusion system at any time *t* is represented by the vector state vector *X*(*t*) = {*X*_1,1_ (*t*), *X*_1,2_(*t*), …, *X*_1,*K*_(*t*),…, *X*_*n*,*k*_(*t*),…,*X*_*N*,*K*_(*t*)}, where *X*_*n*,*k*_(*t*) denotes the molecule population of species *S*_*n*_ in the grid *V*_*k*_ at time *t*. Reactions in each compartment is governed by the Chemical Master Equation (CME), while diffusion is modeled as random walk across neighboring compartments. Each reaction channel *R*_*j*_ in any compartment *k* can be characterized by the *propensity function* *a*_*j*,*k*_ and the *state change vector* *ν*_*j*_≡(*ν*_1*j*_,*ν*_2*j*_,…,*ν*_*Nj*_). The dynamics of the diffusion of species *S*_*i*_ from compartment *V*_*k*_ to *V*_*j*_ is formulated by the *diffusion propensity function* *d*_*i*,*k*,*j*_ and the *diffusion state change vector* *μ*_*k*,*j*_ similarly. *d*_*i*,*k*,*j*_(*x*)*d* *t* gives the probability that, given *X*_*i*,*k*_(*t*)=*x*, one molecule of species *S*_*i*_ at grid *V*_*k*_ diffuses into grid *V*_*j*_ in the next infinitesimal time interval [*t*,*t*+*d* *t*). If *j*=*k*±1, then $d_{i,k,j}(x)=\frac {D}{h^{2}} x$, where *D* is the diffusion rate coefficient and *h* is the characteristic length, also called discretization size, of a grid; Otherwise *d*_*i*,*k*,*j*_=0. The state change vector *μ*_*k*,*j*_ is a vector of length *K* with −1 in the *k*-th position, 1 in the *j*-th position and 0 everywhere else.

With the reaction-diffusion propensity functions and state change vectors, the RDME completely depicts the dynamics of the system:

1 $$  {\begin{aligned} & \frac{\partial P(\mathbf{x},t|\mathbf{x_{0}}, t_{0})}{\partial t} \\ &\quad= {\sum_{k=1}^{K}} {\sum_{j=1}^{M}} \left(a_{j,k}(\mathbf{x}-\nu_{j,k}) P(\mathbf{x}-\nu_{j,k}, t|\mathbf{x_{0}}, t_{0}) -a_{j,k}(\mathbf{x})P(\mathbf{x}, t|\mathbf{x_{0}}, t_{0})\right) \\ & \qquad+ {\sum_{i=1}^{N}\sum_{k=1}^{K}\sum_{j=1}^{K}} \left(-d_{i,k,j}(x_{ik})P(\mathbf{x},t|\mathbf{x_{0}}, t_{0})\right.\\ & \qquad \left. + d_{i,k,j}(X_{ik}-\mu_{k,j}) P(X_{11},\ldots,X_{ik}-\mu_{k,j}, \ldots, X_{N,K}, t| \mathbf{x_{0}}, t_{0})\right), \end{aligned}}  $$

where *P*(**x**,*t*|*x*_0_,*t*_0_) denotes the probability that the system state *X*(*t*)=**x**, given that *X*(*t*_0_)=*x*_0_. The RDME is a set of ODEs that gives one equation for every possible state. It is both theoretically and computationally intractable to solve RDME for practical biochemical systems due to the huge number of possible combinations of states. Instead of solving RDME for the time evolution of the probabilities, we can construct numerical realizations of **X**(*t*). A popular method to construct the trajectories of a reaction-diffusion system is to simulate each diffusive jumping and chemical reaction event explicitly. With enough trajectory realizations, we can derive the distribution of each state vector at different times.

The RDME model have been used as an approximation of the Smoluchowski framework in the mesoscopic scale. Furthermore, researches have discovered that in the microscopic limit, bimolecular reactions may be eventually lost when the grid size becomes infinitely small in the three dimensional domain [21, 23]. The RDME framework requires that the two reactant molecules for a bimolecular reaction must be in the same compartment in order to fire a reaction. Intuitively, we may realize that with more discrete compartments, it is less likely for the two molecules to encounter each other at the same compartment in a high dimensional domain. In order to model the reaction-diffusion system with RDME in the microscopic limit, Radek and Chapman [22] derived a formula of mesh-dependent reaction propensity correction for bimolecular reactions when the discretization size *h* is larger than a critical size *h*_*crit*_. This reaction propensity correction formula fails when the discretization size *h* is smaller than this critical value. Recently, Isaacson [36] proposed a convergent RDME framework (cRDME). In the cRDME framework the diffusion is modeled exactly as in the RDME, while the bimolecular reaction occurs with a nonzero propensity, as long as the distance of the two reactant molecules is less than the reaction radius as defined in the Smoluchowski framework.

In conclusion, the discretization size for the RDME framework should be small enough to avoid discretization error. Yet when the mesh size is less than a critical value, the RDME may become inaccurate for the loss of bimolecular reactions in high dimensional domains. In this paper we will demonstrate that discretization size in space also has great influence on Hill function dynamics in reaction-diffusion systems. The switch-like Hill dynamics breaks even in a 1D domain when the discretization size is small.

### Hill function

The Hill function [7], as well as the Michaelis-Menten function [6] are widely used in enzyme kinetics modeling. In molecular biology, enzymes catalyze biochemical substrates into products, while remaining unchanged. The enzyme kinetics reactions are usually formulated as

2 $$ E+ S\underset{k_{-1}}{\overset{k_1}{\rightleftharpoons }} E S\overset{k_2}{\to } E+ P $$

Leonor Michaelis and Maud Leonora Menten proposed the “quasi-steady state” assumption and formulated the reaction rate equation for the enzyme kinetics, which is mostly referred to as the “Michaelis-Menten” equation. With the conservation law and the quasi-steady state assumption, the Michaelis-Menten equation is given as

3 $$ \frac{d[\!P]}{dt} = V_{max} \frac{[\!S]}{K_{M} + [\!S]},   $$

with *V*_*max*_=*k*_2_[ *E*]_0_ being the maximum reaction rate and $K_{m} = \frac {k_{-1}+k_{2}}{k_{1}}$ being the Michaelis constant.

Sometimes one substrate molecule can have several enzyme binding sites and multiple bindings (cooperative binding) with enzymes are required to activate the substrate.

4 $$ S+ nE\underset{k_{-1}}{\overset{k_1}{\rightleftharpoons }}{SE}_n\overset{k_2}{\to } nE+ P $$

In real biological models, the binding of the *n* enzyme molecules to a substrate does not take place at once but in a succession of steps. Using the quasi-steady state assumption and conservation laws, the Hill function that formulates the reaction dynamics is given as

5 $$ \frac{d[\!P]}{dt} = V_{max} \frac{[\!E]^{n}}{{K_{m}}^{n} + [\!E]^{n}},   $$

with *V*_*max*_ as the maximum reaction rate, *K*_*m*_ as the Michaelis constant, and *n* as the Hill coefficient. The Hill function is widely used to model “step-regulated” reaction as an activity switch.

## Results

**Fig. 2:**
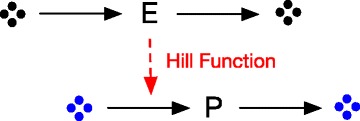
A simple toy model of Hill function dynamics in 1D domain. Enzyme *E* is constantly synthesized and upregulates the synthesis of product *P*

To simplify the analysis, a toy model of a reaction-diffusion system in one dimension is constructed. As demonstrated in Fig. 2, in the toy model an enzyme species *E* (typically a transcription factor) is constantly synthesized and degraded. The enzyme *E* further upregulates the DNA expression of a product *P*. The synthesis rate of *P* is formulated as a Hill function.

Assume a spatial domain of size *L* is equally partitioned into *K* compartments with size *h*=*L*/*K* for each. The list of reactions and reaction propensities in each compartment are given as

6 $$  \begin{array}{rcl rcl} \emptyset &\to & E_{i}, & a_{1} &=& k_{s} \cdot h ;\\ E_{i} & \to & \emptyset, & a_{2} &=& k_{d} \cdot E_{i} ;\\ \emptyset & \xrightarrow{E_{i}} & P_{i}, & a_{3} &=& k_{syn}\cdot h \frac{E_{i}^{4}}{(K_{m}\cdot h)^{4} + E_{i}^{4}};\\ P_{i} &\to & \emptyset, & a_{4} & = & k_{deg} \cdot P_{i};\\ E_{i} &\to & E_{i\pm 1}, & a_{5} &= &2\frac{D_{E}}{h^{2}} E_{i}; \\ P_{i} &\to & P_{i\pm 1}, & a_{6} &= &2\frac{D_{P}}{h^{2}} P_{i}; \\ \end{array}  $$

The parameters *k*_*s*_, *k*_*d*_ are the synthesis, degradation rates, respectively, for enzyme species *E*, and similarly *k*_*syn*_, *k*_*deg*_ are those for product *P*. *K*_*m*_ is the Michaelis constant in the Hill function.

In the one-dimensional domain, the enzyme *E* is constantly synthesized and degraded. At the equilibrium state, the distribution of the total population of *E* is given by the Poisson distribution,

7 $$ P_{E}(n) = \frac{\alpha^{n}}{n!} e^{-\alpha},  $$

where $\alpha = \frac {k_{s}}{k_{d}}L$ denotes the mean value of the total number of enzyme *E* molecules in the domain.

For an individual compartment (bin), consider the probability $P_{E}^{(i)}(n)$ that an individual bin *i* contains *n* molecules of enzyme *E*. At the equilibrium state, enzyme *E* is homogeneously distributed in the system. The probability that each molecule of *E* stays in a certain bin *i* is given by *p*=1/*K*. The probability that, of all the *E* molecules in the domain, none is in bin *i* is approximated by

8 $$ \begin{array}{rcl} P_{E}^{(i)}(0) & = & P_{E}(0) + P_{E}(1)(1-\frac{1}{K}) + P_{E}(2)(1-\frac{1}{K})^{2}\\ &&+ \ldots +P_{E}(N)(1-\frac{1}{K})^{N} + \ldots\\ & = & {\sum\limits_{n=0}^{N}e^{-\alpha} \frac{\alpha^{n}}{n!} \left(1-\frac{1}{K}\right)^{n}} \\ & = & e^{-\alpha/K}. \end{array}  $$

The other probability terms are not important in the analysis.

With the distribution of the enzyme molecular population, the mean reaction propensity for the synthesis of protein *P* in the *i*-th bin is

9 $$ \langle a^{i}_{syn} \rangle = k_{syn} h \sum_{n=0}^{\infty} \frac{n^{4}}{(K_{m}\cdot h)^{4}+ n^{4} }P_{E}^{(i)}(n).  $$

Notice that when *n*=0, the Hill function is zero, and when the discrete bin size *h* is small, the Hill function approaches one quickly if *n*≥1. For example, when *K*_*m*_·*h*≤0.5 the Hill function $\frac {n^{4}}{(K_{m}\cdot h)^{4} + n^{4}} \ge 0.94$ for *n*≥1. Therefore, upper and lower bounds for the product *P* synthesis propensity, when *k*_*m*_·*h*≤0.5, are

10 $$ 0.94 k_{syn}\cdot h \sum_{n=1}^{\infty} P_{E}^{(i)}(n) \le \langle a^{syn} \rangle \le k_{syn}\cdot h \sum\limits_{n=1}^{\infty} P_{E}^{(i)}(n).  $$

Hence, when the discretization size *h* is small enough, the propensity for the product *P* synthesis reaction can be approximated as

11 $$ \begin{array}{rcl} \langle a_{syn}^{(i)} \rangle & \approx & k_{syn}\cdot h \cdot {\sum\limits_{n=1}^{\infty} P_{E}^{(i)}(n)}\\ & = & k_{syn} \cdot h \cdot (1 - P_{E}^{(i)}(0))\\ & = & k_{syn} \cdot h \cdot (1 - e^{-\alpha/K}). \end{array}  $$

When the discretization size *h* is small and *K* is large, the mean reaction propensity can be further approximated as

12 $$ \langle a_{syn}^{(i)} \rangle \approx k_{syn} \cdot h \cdot \alpha / K.   $$

Notice that *α*/*K* is the mean population of enzyme *E* in the *i*-th bin. The Hill function of the product *P* synthesis is now reduced to a *linear function* of the enzyme *E* population in the *i*-th bin.

Furthermore, from (12) the mean population of product *P* in the bin *i* is

13 $$ \langle P^{(i)} \rangle = \frac{k_{syn}\cdot h}{k_{deg}}\frac{\alpha}{K},  $$

and the total product *P* population in all *K* bins is

14 $$ \begin{array}{rcl} \langle P \rangle &=& {\frac{k_{syn} \cdot L}{k_{deg}}\frac{k_{s}\cdot L}{k_{d}}\frac{1}{K}}\\ & = & {\frac{k_{syn}}{k_{deg}}\cdot \alpha \cdot h}. \end{array}   $$

**Fig. 3:**
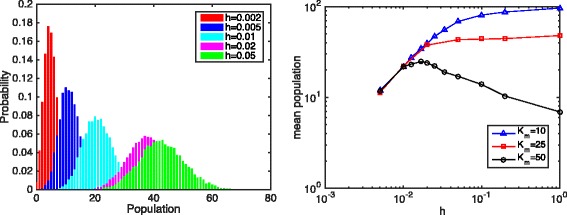
The histogram (*left*) and mean (*right*) population of product *P* with different discretization. Parameters: *D*_*e*_=1.0, *k*_*s*_=2.5, *k*_*d*_=0.1, *k*_*syn*_=5.0, *k*_*deg*_=0.05, system size *L*=1.0. For the histogram figure, *K*_*m*_=25.0. The log-log plot shows the mean total product population under different discretization and different parameter sets

Equation 14 shows that the total population of product *P* is a linear function of *α*, the mean population of *E* and *h*=*L*/*K*, the discretization size. With finer discretization, less product *P* is produced. Figure 3 shows the histograms and the mean values of the product *P* population with different discretization sizes. The histograms show that with finer discretization, the population histograms shift further to the left.

The log-log plot (Fig. 3, right) shows that when the discretization size is small enough, the total product *P* population is a linear function of discretization size. The slope of the log-log plot is about 1.0 at small discretization size *h*, regardless of *K*_*m*_.

Moreover, simulation results show that when the mean enzyme *E* population is less than the constant *K*_*m*_ in the Hill function (*K*_*m*_>*α*), the population of product *P* increases slightly before the Hill function dynamics breaks at small discretization sizes. Note that the Hill function dynamics show a concave shape with respect to enzyme *E* population when the enzyme *E* population is smaller than the Michaelis constant *K*_*m*_. Therefore, it is reasonable that the product *P* population in this reaction-diffusion model increases slightly when the Michaelis constant *K*_*m*_ is larger than the mean enzyme *E* population *α*.

The numerical analysis above makes two approximations

15 $$ \left\{\begin{array}{l} \frac{n^{4}}{(K_{m}\cdot h)^{4} + n^{4}} \approx 1, \text{for}\,n \ge 1;\\ e^{-\alpha/K} \approx 1 - \alpha/K. \end{array}\right.  $$

to get the linear relation. Assuming an error tolerance of 5%, the two approximations can be simplified to

16 $$ \left\{\begin{array}{l} K_{m}\cdot h < 0.5,\\ \alpha/K < 1/3. \end{array}\right.   $$

Hence, when the discretization bin number

17 $$ K > \max\{2{LK}_{m}, \quad 3\alpha \},  $$

the Hill dynamics reduce to a linear function.

Equivalently, in order for the Hill function dynamics to work well, the discretization number *K* should be less than or equal to this threshold. However, the coarse discretization from a small *K* leads to spatial error. Two potential solutions to this discretization dilemma are proposed next.

## Discussion

From the previous analysis, the Hill dynamics in RDME systems reduces to a linear function due to the lack of intermediate states — the discrete population in each individual bin yields an integer value (0 or 1) for the Hill function. Thus a natural solution to it is to generate intermediate states by a smoothing technique that averages the population over neighboring bins when calculating the reaction propensity.

To model a RDME system in high dimensions with fine discretization, previous studies [21] have suggested relaxing the same-compartment reaction assumption and allowing reactions within neighboring compartments. The next subsection shows that allowing reactions within neighboring compartments is equivalent to smoothing over neighboring compartments.

### Smooth over neighboring bins

A natural technique that bridges the discrete and continuous models is to smooth the spatial population by taking the average of neighboring bins. Consider first smoothing the enzyme *E* population within the neighboring *m* bins (including the bin itself) when calculating the reaction propensity.

Following previous analysis, the reaction probability for the synthesis of product *P* in the *i*-th bin is

18 $$ \begin{array}{rcl} \langle \hat{a}_{syn}^{(i)} \rangle & = & k_{syn}\cdot h {\sum\limits_{\hat{n} = 0}^{\infty} \left(\frac{(n/m)^{4}}{(K_{m}\cdot h)^{4} + (n/m)^{4}}P_{E}^{(i)}(n; m)\right)} \\ & = & k_{syn}\cdot h {\sum\limits_{n = 0}^{\infty} \left(\frac{n^{4}}{(m\cdot K_{m}\cdot h)^{4} + n^{4}}P_{E}^{(i)}(n; m)\right)}, \end{array}   $$

where $P_{E}^{(i)}(n; m)$ denotes the probability that the *m* neighboring bins of the *i*-th bin have a total enzyme *E* population of *n*. The interpretation of this equation is that the synthesis reaction in the *i*-th bin is interacting with the *m* neighboring bins and the propensity is calculated based on the total enzyme *E* population of all the neighboring bins. By probability theory,

19 $$ P_{E}^{(i)}(0; m) = e^{-\alpha m/K}.  $$

As before, only the term ${P}_{E}^{(i)}(0; m)$ is important.

In Eq. (18), for any fixed integer *m*≥0, there exists an *h*≥0, such that *m*·*K*_*m*_·*h*<0.5 and the Hill function is still approximately one. With such a discretization size *h*, the product *P* synthesis propensity can be approximated as

20 $$ \begin{array}{rcl} \langle \hat{a}^{(i)}_{syn} \rangle &\approx & k_{syn}\cdot h {\sum\limits_{n = 1}^{\infty} P_{E}^{(i)}(n; m)}, \\ & = & k_{syn}\cdot h (1 - P_{E}^{(i)}(0; m)), \\ & = & k_{syn}\cdot h (1 - e^{-\alpha m/K}) \\ & \approx & k_{syn}\cdot h \cdot \alpha \cdot m/K. \end{array}  $$

Again, with a fixed smoothing bin number *m*, the synthesis reaction propensity becomes linear in the mean enzyme *E* population *α* *m*/*K* of the *m* bins, and the mean population of product *P* in the system is

21 $$ \langle P \rangle = \frac{k_{syn} \cdot L}{k_{deg}}\frac{k_{s}\cdot L}{k_{d}}\frac{m}{K},   $$

which is linear in *m*/*K* and the mean total enzyme *E* population *α*. The linear function can be achieved with an *h* such that

22 $$  \begin{cases} m\cdot K_{m}\cdot h < 0.5,\\ m\cdot \alpha/K < 0.33. \end{cases}  $$

**Fig. 4:**
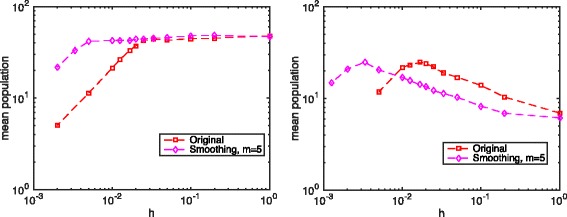
The total population of product P with different discretization. Parameters: system size *L*=1.0, *D*_*e*_=1.0, *k*_*s*_=2.5, *k*_*d*_=0.1, *k*_*syn*_=5.0, *k*_*deg*_=0.05. For the left figure *K*_*m*_=25.0, while for the right figure *K*_*m*_=50.0

Figure 4 plots the mean population of product *P* in the toy model with the smoothing technique and *m*=5. Numerical results show that smoothing over a fixed number *m* of compartments gives a good solution for a certain range of discretization sizes. However, there always exists a small enough critical discretization size *h*_*crit*_ such that the Hill function dynamics reduce to a linear function when the discretization size is smaller than this *h*_*crit*_. Moreover, fixed length smoothing, in the scenarios where the Michaelis constant *K*_*m*_ is larger than the mean enzyme *E* population *α*, gives a result closer to that of the deterministic simulation when the discretization sizes are not too small.

### Convergent hill function dynamics in reaction-diffusion systems

The previous subsection demonstrates that a sufficiently small discretization size *h* will still break the Hill dynamics even with the strategy of smoothing over a fixed number of bins, thus the number of bins needs to vary with the discretization size.

Inspired by the convergent-RDME framework [36], a remedy for the failure of Hill function dynamics in reaction-diffusion systems is to smooth the population over bins within a certain distance.

From the analysis, a small smoothing length would cause the failure of the Hill function dynamics and a large smoothing length would degrade the spatial accuracy of the model. Based on the criteria of failure for the Hill function dynamics with fixed *m*, Eq. (22), we can choose the smallest *m* that would not result in failure for the Hill function dynamics, i.e., *m* such that neither of the two assumptions in the previous analysis are valid. This choice is

23 $$  m = \lceil \max\left\{\frac{0.5}{K_{m}\cdot h}, \frac{0.33\cdot L}{\alpha \cdot h}\right\} \rceil.  $$

Following the terminology in the convergent-RDME framework [36], the “reaction radius *ρ*” of the Hill function dynamics is defined as *ρ*=*m*·*h*, where *m* is given in (23).

**Fig. 5:**
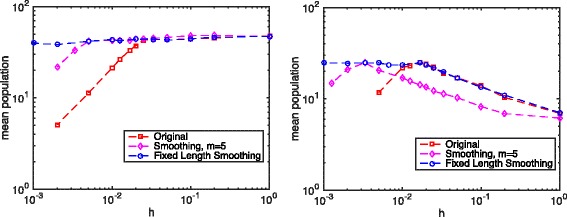
The total population of product P with different discretization. Parameters: system size *L*=1.0, *D*_*e*_=1.0, *k*_*s*_=2.5, *k*_*d*_=0.1, *k*_*syn*_=5.0, *k*_*deg*_=0.05. For the left figure *K*_*m*_=25.0, while for the right figure *K*_*m*_=50.0

Figure 5 shows numerical results for the toy model in the reaction-diffusion system with different discretization sizes and with the convergent smoothing technique (*m* and *h* related by (23)). It is clear that the convergent smoothing technique gives very good simulation results for all *h* values.

**Fig. 6:**
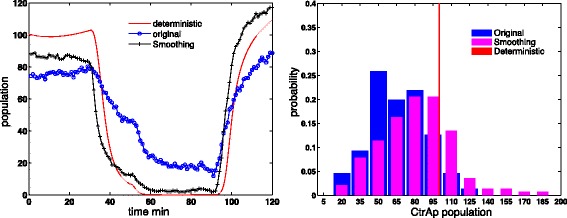
The Comparison of CtrAp of deterministic model and the stochastic simulation results. *Left*: CtrAp population oscillation trajectory during *Caulobacter crescentus* cell cycle. *Right*: The histogram of CtrAp population in the swarmer cells (*t*=30*m* *i* *n*). For model parameters, please refer to [27]

Applying the fixed length smoothing technique to the DivL-CckA Hill function model in the *Caulobacter crescentus* cell cycle results in a sharp CtrAp population change during swarmer-to-stalked transition. Figure 6 shows the CtrAp trajectories from the deterministic model and stochastic model simulation results. The fixed length smoothing technique yields more CtrAp in the swarmer stage and less CtrAp in the stalked stage, which yields a sharp CtrAp population change during the swarmer-to-stalked transition as expected.

## Conclusions

Motivated by the misbehavior of DivL-CckA dynamics in the stochastic simulation of the *Caulobacter crescentus* cell cycle model, a study of the Hill function dynamics in reaction-diffusion systems reveals that when the discretization size is small enough, the switch-like behavior of Hill function dynamics reduces to a linear function of input signal and discretization size. A proposed fixed length smoothing method, which allows chemical reactions to occur with reactant molecules within a distance of fixed length, the “reaction radius”of the Hill function dynamics, seems to give a very good remedy to this problem.

It is known that in high dimensions bimolecular reactions are lost with the RDME in the microscopic limit [21]. This work shows that one-dimensional Hill function dynamics in a RDME framework gives a similar challenge when the discretization size is small enough. The conjecture is that the problem lies in the RDME requirement that reactions only fire with the reactant molecules in the same discrete compartment.

Furthermore, this defect in RDME at the microscopic limit is believed to be a common scenario for all highly nonlinear reaction dynamics. Theoretical biologists have developed many highly nonlinear reaction dynamics that need special attention when converted to stochastic models.

Here we will extend our analysis and discuss a general situation in stochastic simulation of reaction diffusion systems. Suppose that we have a species *X*, whose population is represented by state variable *x*, and there is a particular reaction *R*:

24 $$  \emptyset \mathop{\longrightarrow}^{X} P,  $$

in which *X* serves as an enzyme to produce *P* and the propensity function is represented by *f*(*x*). For each *X* molecular, it can diffuse in a 1D domain with a small length *L* and with a diffusion coefficient *D*. Suppose the 1D domain is partitioned into *K* bins, thus the discretization size is $h = \frac {L}{K}$. The system can then be represented as a chain reaction

25 $$  X_{1} \mathop{\rightleftharpoons}^{d}_{d} X_{2} \mathop{\rightleftharpoons}^{d}_{d} \cdots \mathop{\rightleftharpoons}^{d}_{d} X_{K},  $$

where $d = \frac {D}{h^{2}}$ is the jump rate corresponding to diffusion. The concerned reaction *R* could fire in any of the bins with propensity *f*(*x*_*i*_). Assume that *L* is small enough such that $\frac {D}{L^{2}}$ is very large and $d \gg \sum _{i=1}^{K} f(x_{i})$ regardless of *K*. In that case, the chain reaction system (25) can be considered as a virtual fast system and the slow scale SSA [37] can be applied here. As a result, if the total population of *X* is *n*, in each bin, the mean value of *x*_*i*_ is given by

26 $$ \langle x_{i} \rangle = \frac{n}{K}.  $$

Then based on the theory of slow scale SSA, the propensity of the corresponding synthesis reaction (24) should be

27 $$ \langle f(x_{i}) \rangle = \sum_{j=0}^{\infty} f(j) P(x_{i} = j),  $$

where *P*(·) is the probability under the distribution when the virtual fast system (25) converges to stochastic partial equilibrium [37].

However, the propensity function converted directly from the deterministic model has a different form as *f*(〈*x*_*i*_〉). Note that for a nonlinear function, such as the Hill function or the Michaelis Menten function,

28 $$  \langle f(x_{i}) \rangle \neq f\left(\langle x_{i} \rangle \right).  $$

(28) highlights the mismatch between the RDME framework and the deterministic model.

### Hybrid method

In order to have a stochastic model that is consistent with its deterministic counterpart, the propensity function should take the form *f*(〈*x*_*i*_〉). This motivates us to adopt the hybrid ODE/SSA method [38] and apply it to the reaction diffusion systems. This hybrid method was a simple idea. It was originally presented by Haseltine and Rawlings [38] and our implementation has some modification to make it fit better with the root finding function used in LSODAR [39]. Consider a system of *N* species (denoted by {*S*_1_,…,*S*_*N*_}) and *M* reactions (denoted by {*R*_1_,…,*R*_*M*_}). For each reaction *R*_*j*_, there is a propensity function *a*_*j*_(*x*) and a state-change vector *ν*_*j*_. We partition these *M* reactions into two subsets. The subset *S*_*slow*_ contains slow reactions, with index 1 to *M*_*S*_, and is simulated by the SSA. The subset *S*_*fast*_ contains fast reactions, with index *M*_*S*_+1 to *M*, and is formulated and solved by ODEs. The simulation of these two subsets is then combined as described below. Let *τ* be the jump interval of the next slow (stochastic) reaction, and *μ* be its reaction index. Set *t*=0. The hybrid method simulate the system as follows:

- Two uniform random numbers, *r*_1_ and *r*_2_ in *U*(0,1), are generated.
- Solve the ODE system for *S*_*fast*_ and find the root *τ* for the integral equation:
  29 $$ \int^{t+\tau}_{t} a_{tot}(\mathbf{x},s)ds+\log(r_{1}) = 0,   $$
  where *a*_*tot*_(**x**,*t*) is the sum of propensities of all reactions in *S*_*slow*_. Because **x** varies with *t* in the ODE system, *a*_*tot*_(**x**,*t*) is a function of *t* as well.
- *μ* is selected as the smallest integer satisfying
  30 $$ \sum^{\mu}_{i=1}a_{i}(\mathbf{x},t) > r_{2} a_{tot}(\mathbf{x},t).   $$
- Update $\mathbf {x}\leftarrow \mathbf {x}+\mathbf {\nu }_{\mu }$.
- Return to step 1) if stopping condition is not reached.

Note that our implementation is different from Haseltine and Rawling’s original method in step 2. Suppose that the ODE system is given by

31 $$  \mathbf{x}'=f(\mathbf{x}).  $$

We add an integration variable *z* and the following equation to the ODE system.

32 $$  z'=a_{tot}(\mathbf{x}), \quad z(t)=\log(r_{1}),  $$

where we note that log(*r*_1_) is negative and *a*_*tot*_ is always nonnegative. In the hybrid simulation, for each step we start from the current time *t* and numerically [39] integrate the original ODEs (31) and the extra integral Eq. (32). The integration stops when *z*(*t*+*τ*)=0. As a result, *τ* is the solution to (29). This procedure can be numerically simulated using standard ODE solvers combined with root-finding functions, such as the LSODAR [39]. Note that since *z* is an integration variable, one may choose to omit it from the error control mechanism [40]. Adding this extra variable will not greatly affect the efficiency.

**Fig. 7:**
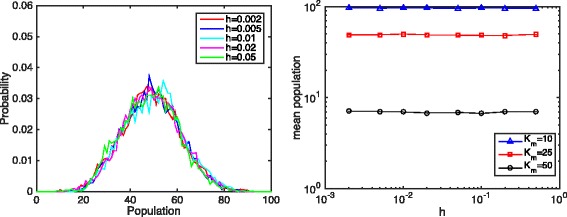
The histogram (*left*) and mean (*right*) population of product *P* with different discretization, simulated by the hybrid method. Parameters: *D*_*e*_=1.0, *k*_*s*_=2.5, *k*_*d*_=0.1, *k*_*syn*_=5.0, *k*_*deg*_=0.05, system size *L*=1.0. For the histogram figure, *K*_*m*_=25.0. The log-log plot shows the mean total product population under different discretization and different parameter sets

**Fig. 8:**
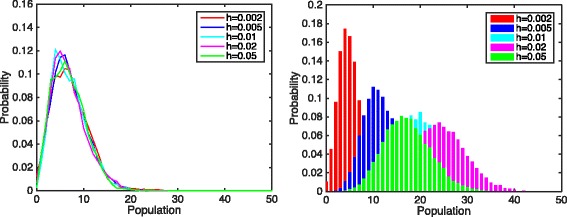
The distribution of product *P* with different discretization sizes, simulated by the hybrid method (*left*) and SSA (*right*). Parameters: *K*_*m*_=50.0, the rest remains the same

We applied the hybrid method to the toy model (6). In our simulation, all diffusion events are partitioned into fast systems and solved by the ODE solver LSODAR, while chemical reactions are simulated by SSA under the hybrid framework described above. We test cases when *K*_*m*_=10,25,50 and Figs. 7 and 8 show the corresponding numerical results. It is obvious that in all three cases, the mean population remains horizontal even when the bin size decreased to the magnitude of 10^−3^. In Fig. 8, the mean molecule of product *P* centers around seven under different discretization sizes, while results from SSA shift to the left as discretization size decreases.

Numerical results certainly suggest that the hybrid method has great potential in stochastic simulation of RD systems. We would like to note that great details still need to be studied, but that is not the focus for this paper.

## Abbreviations

CME: Chemical master equation
ODE: Ordinary differential equation
PDE: Partial differential equation
RDME: Reaction diffusion master equation
SSA: Stochastic simulation algorithm
